# Seroprevalences of Newly Discovered Porcine Pestiviruses in German Pig Farms

**DOI:** 10.3390/vetsci6040086

**Published:** 2019-10-25

**Authors:** Anna Michelitsch, Anja Dalmann, Kerstin Wernike, Ilona Reimann, Martin Beer

**Affiliations:** Institute of Diagnostic Virology, Friedrich-Loeffler-Institut, 17493 Greifswald—Insel Riems, Germany; Anna.Michelitsch@fli.de (A.M.); Anja.Dalmann@fli.de (A.D.); Ilona.Reimann@fli.de (I.R.); Martin.Beer@fli.de (M.B.)

**Keywords:** pestiviruses, Bungowannah virus, atypical porcine pestivirus, serology, swine, prevalence, epidemiology

## Abstract

Several novel porcine pestiviruses that are linked to disease outbreaks in commercial pig farms were discovered during recent years. Bungowannah pestivirus (BuPV; new species *Pestivirus F*) causes sudden death in young pigs, but has only ever been isolated in the Australian region Bungowannah. Atypical porcine pestivirus (APPV; new species *Pestivirus K*) on the other hand has been found in multiple countries worldwide and is potentially linked to congenital tremor, a disease that causes considerable production problems in pig farms. To assess the seroprevalences of both viruses in German commercial farms during the years 2009/10 and 2018, two approaches were selected. Antibodies against *Pestivirus F* were detected by a traditional in-house indirect immunofluorescence test against the culture-grown virus isolate, while for the detection of *Pestivirus K*-specific antibodies, a newly developed test system utilizing a chimeric construct of bovine viral diarrhea virus 1 (BVDV-1; species *Pestivirus A*) containing the E1 and E2 encoding sequences of APPV was established. A total of 1115 samples originating from 122 farms located in seven German federal states were investigated. Antibodies against Bungowannah virus could not be detected, confirming the absence of this virus in other regions than the initially affected Australian pig farm complex. In contrast, antibodies against APPV were highly prevalent throughout Germany at both investigated time points. The seroprevalence at the state level fluctuated to some degree, but the overall percentage remained stable, as is to be expected for an endemic pestivirus lacking any form of control measures.

## 1. Introduction

Pestiviruses (family Flaviviridae) are a group of viruses of veterinary importance that infect cloven-hoofed animals like pigs, wild boar, cattle, sheep, and different deer species, thereby causing major economic losses in livestock animals. While for decades only four classical species, i.e., classical swine fever virus (CSFV), bovine viral diarrhea virus types 1 (BVDV-1) and 2 (BVDV-2), border disease virus (BDV), and a few so-called atypical pestiviruses were known, the phylogenetic and host diversity of the genus *Pestivirus* expanded considerably during recent years (reviewed in [1]). Due to the high number of newly discovered virus species, the genus was recently reorganized [2]. While the classical pestivirus species BVDV-1, BVDV-2, CSFV, and BDV are now referred to as *Pestivirus A*, *B*, *C*, and *D*, respectively, the newly discovered viruses form the species *E* to *K* [3]. Most of these new viruses were found during survey studies aimed at the identification of known pestiviruses in further ungulate species as possible hosts [4,5] or by untargeted high-throughput sequencing approaches of bats or rats, which are known for their potential to transmit pathogenic agents [6,7]. However, connections between virus detection and disease outbreaks could often not be established. In contrast, the first of the novel porcine pestiviruses was isolated from a pig farm in Australia in 2003 during an outbreak of sudden death in young pigs, followed by an increase in stillbirth [8], and the observed clinical signs could be connected to the newly detected virus [9,10]. Tentatively named Bungowannah virus (BuPV), according to the farm of its initial detection, the virus belongs to the new species *Pestivirus F*, and although it is a potential threat to commercial pig farming, it has not yet been reported from any other farm, region, or country [11].

Another porcine pestivirus was discovered in North America in 2015 [12] and was, until now, also found in South America, Canada, Europe, and Asia [13,14,15,16,17,18,19]. Named as atypical porcine pestivirus (APPV), this species is now classified as *Pestivirus K* [3]. *Pestivirus K* is supposed to be linked to congenital tremor (CT) [19,20], a disease that leads to myoclonic tremors in suckling pigs, hindering the consumption of milk and, therefore, often resulting in death of those animals [21]. With type A-I congenital tremor being caused by classical swine fever virus, type A-III and A-IV originating from genetic defects, and type A-V caused by intoxication with trichlorfon, type A-II was suspected to be caused by some type of virus long before the detection of this new pestivirus [20]. During the search for the causative agent of type A-II CT, another pestivirus was found in Austria and provisionally named LINDA virus (lateral-shaking inducing neurodegenerative agent). LINDA virus interestingly showed a higher sequence identity to BuPV than to APPV [22].

Until now, the research on these newly discovered ‘atypical’ porcine pestiviruses has mainly focused on the detection of virus genomes and sequences, proving the occurrence of *Pestivirus K* in a variety of countries and LINDA virus in Austria, but has failed to detect the overall prevalence and infectious burden in commercial pig farms, which can be achieved by antibody prevalence studies. While RT-PCR is a useful tool to detect the presence of actual virus genomes, a serological test for antibody detection can give further important information about the overall contact of animals with the virus in question. However, the usage of commercially available antibody-ELISAs, which were originally developed for the detection of antibodies against classical pestiviruses, is not usable for the newly discovered atypical pestiviruses due to a lack of cross-reactivity [23].

In the present study, a first regional study on the antibody prevalence against members of the species *Pestiviruses F* and *K* was conducted in Germany, a country with massive pig production and, therefore, a high interest in awareness of potential threats to production. For the detection of antibodies against BuPV, an indirect immunofluorescence test based on the original virus isolate was established, while for APPV, a chimeric pestivirus was constructed, circumventing the need to isolate newly detected pestiviruses before serology-based prevalence studies can be conducted.

## 2. Materials and Methods

### 2.1. Diagnostic Samples

A total of 1115 porcine serum samples originating from 122 farms located in seven German states (Bavaria (BY), Baden-Wuerttemberg (BW), North Rhine-Westphalia (NW), Lower-Saxony (NI), Saxony-Anhalt (ST), Brandenburg (BB), and Mecklenburg-Western Pomerania (MV)) were used. Six hundred of them were collected during the years 2009 or 2010, while the remaining 515 sera were collected in 2018. The numbers of herds and individual samples per federal state are given in Table 1.

The samples were kindly provided by the German local diagnostic laboratories. All samples were taken by the responsible farm veterinarian during routine testing in the context of the health-monitoring program of the farms, therefore information about age, sex and health status of the animals is not available. However, none of the samples originated from suckling piglets.

### 2.2. Construction of the Plasmid pA/CP7_E1E2_APPV

**Figure 1:**
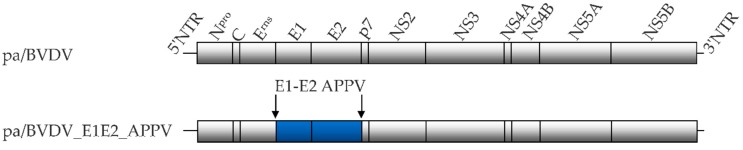
The genome structure of the parental full-length cDNA clone pA/BVDV and the generated chimeric construct pa/CP7_E1E2_APPV. Blue-colored boxes represent the substituted APPV structural proteins E1 and E2. Lines at the left and right ends demonstrate non-translated regions (NTRs).

The pestivirus chimeric construct pA/CP7_E1E2_APPV was generated on the basis of the infectious BVDV-1 cDNA clone pA/CP7 [24]. In this backbone, the E1 and E2 encoding sequences were replaced with that of a selected APPV (GenBank accession number KR011347) (Figure 1) by a modified fusion PCR [25]. In brief, the genomic region encoding APPV-E1 and -E2 was amplified by RT-PCR from the synthetic open reading frame APPV_E1E2p7 APPV_pMK-RQ (Geneart AG, Regensburg, Germany) by using Phusion High-Fidelity DNA Polymerase (New England Biolabs, Frankfurt am Main, Germany) and appropriate primers (biomers.net GmbH, Ulm, Germany). Subsequently, the purified amplicon was used as a megaprimer in a fusion PCR with plasmid pA/CP7 as template. Primers are listed in the Supplementary Materials (Table S1). Details for generation of the recombinant construct are available upon request.

### 2.3. Indirect Immunofluorescence Test

Porcine kidney cells (PK15, cell line 0005-1, Collection of Cell Lines in Veterinary Medicine (CCLV), Insel Riems, Germany), cultivated in Dulbecco’s modified Eagle’s medium (DMEM), supplemented with 10% BVDV-free fetal calf serum, were seeded into 96-well multiplates. After 16 to 18 h of incubation at a temperature of 37 °C, a humidity of 2.5%, and a CO_2_ concentration of 5%, each second row of wells was infected with a 200 tissue culture infectious dose of 50% per well of *Pestivirus F* isolate ‘BuPV’ [8] and incubated at the aforementioned conditions for 24 h. The remaining wells were left uninfected. After the incubation period, the medium was removed and all plates were heat fixated at 80 °C for 2 h.

Bovine oesophageal cells (KOP-R, cell line 0224, CCLV, Insel Riems, Germany), cultivated in modified Eagle’s medium (MEM) supplemented with 10% BVDV-free fetal calf serum, were seeded into 96-well multiplates and incubated with the same parameters as the PK15 cells for 16 to 18 h. Thereafter, each second row of wells was transfected with the chimeric construct pa/CP7_E1E2-APPV using electroporation as described previously [25]. Subsequent to an incubation period of 72 h, the cells were fixed by heat (80 °C, 2 h) and successful replication after transfection was proven by immunofluorescence staining of the BVDV-1 backbone with the pan-pesti reactive monoclonal antibody WB 103/105 (anti-NS3 panpesti, CVL, Weybridge, UK) in one well per transfection batch. The efficiency of transfection was about 50%, allowing the differentiation of transfected cells from nontransfected cells in the examined cell layer, also ensuring, together with the other controls, a high specificity of the read-out.

**Figure 2:**
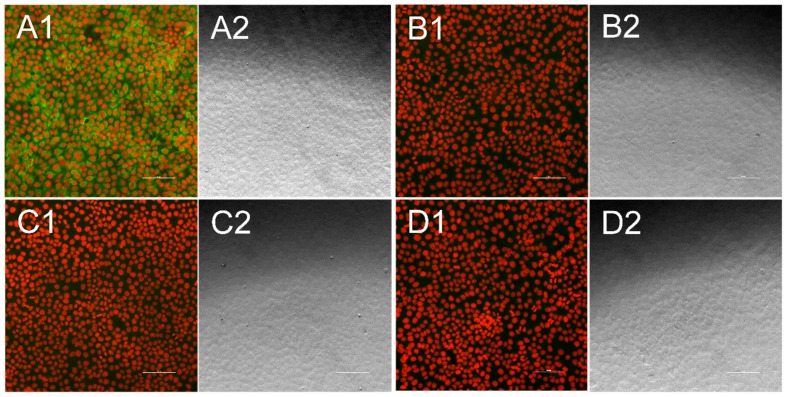
Indirect immunofluorescence test for Bungowannah pestivirus (BuPV)-specific antibodies. Each fluorescence picture (1) is presented alongside a bright field picture, that was taken at the same position (2). Cell nuclei appear in red, while antibodies against BuPV are stained in green. When antibodies were present in the sample they bound to PK15 cells infected with the BuPV isolate (**A**), while no green fluorescence could be seen when antibody-positive samples were tested against uninfected cells (**B**). Antibody-negative serum sample neither showed significant interaction with infected cells (**C**) nor with uninfected cells (**D**). Bar is 100 µm.

**Figure 3:**
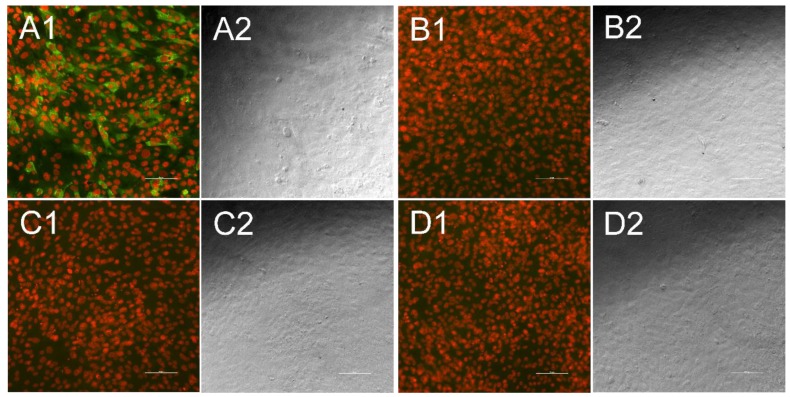
Indirect immunofluorescence test for atypical porcine pestivirus (APPV)-specific antibodies. Each fluorescence picture (1) is presented alongside a bright field picture, that was taken at the same position (2). Cell nuclei appear in red, while antibodies against APPV are stained in green. When antibodies were present in the sample they bound to KOP-R cells transfected with the chimeric cDNA clone pA/CP7_E1E2-APPV (**A**), while no green fluorescence could be seen when antibody-positive samples were tested against untransfected cells (**B**). Antibody-negative serum samples neither showed significant interaction with transfected cells (**C**) nor with untransfected cells (**D**). Bar is 100 µm.

Serum samples were diluted in TBST (Tris-buffered saline, with Tween^®^m20, ph 8.0 powder, Sigma-Aldrich, Steinheim, Germany) in a ratio of 1/100, 1/400, as well as 1/1600 for BuPV testing and 1/100 as well as 1/200 for APPV testing. The dilution ratio was determined by titrating the positive control serum for each virus in duplicates, starting at 1:100, a dilution that is high enough to avoid a background signal that is too intense. Titration was performed in a two-fold dilution series until a 1:12800 dilution was reached. The dilutions that showed a positive signal for the positive control serum were than used for testing. Fifty µL of each dilution was incubated on an infected well, as well as on the neighboring noninfected well for 1 h at room temperature. Following a washing step with TBST, all wells were incubated for 1 h with an FITC (fluoresceinisothiocyanate)-labelled anti swine IgG antibody (VEB Kombinat Veterinärimpfstoffe, Dessau, Germany). Thereafter, the plates were washed three times with TBST and DABCO (1,4-diazabicyclo[2.2.2]octan, Sigma-Aldrich, Steinheim, Germany) fluorescence preservation buffer combined with propidium iodide (Sigma-Aldrich, Steinheim, Germany), to stain cell nuclei, was added. After a 10 min incubation period, plates were analyzed using the 10 × objective lens of the Nikon Eclipse Ti-S inverse fluorescence microscope. The indirect immunofluorescence test is a standard method in virological diagnostics for the detection of antibodies. The evaluation is performed by the human eye. Sera were considered as positive when specific green fluorescence signals were visible gathered around the nuclei, which were stained red and made the cell bodies visible (Figure 2 and Figure 3). As a negative control, the same protocol was used for each sample on noninfected/nontransfected cells and compared to the infected/transfected stains to avoid any false positive evaluation due to possible side interactions between serum sample, cells, and staining antibody. As a further control measure, a positive control serum was tested on each manufactured plate to ensure a consistent quality. The control serum for BuPV tested originated from the outbreak in Australia and the control serum for APPV testing was defined during a screening of reference sera from the Friedrich-Loeffler-Institut.

In order to estimate the antibody titers that are detected with this newly developed method, titration was performed as described above for the control samples, using 37 serum samples that tested positive for APPV.

**Figure 4:**
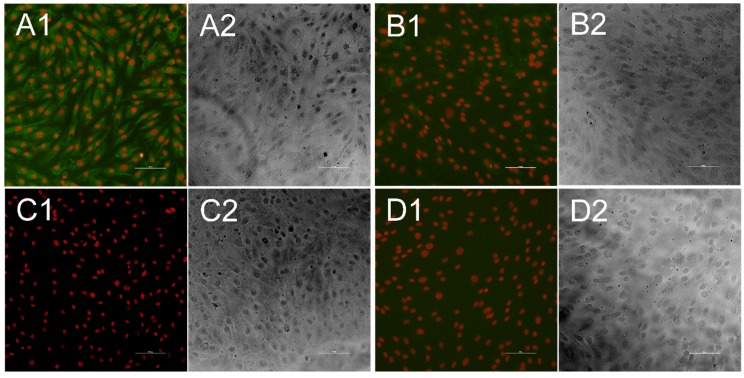
Indirect immunofluorescence test for bovine viral diarrhea virus 1 (BVDV)-specific antibodies. Each fluorescence picture (1) is presented alongside a bright field picture, that was taken at the same position (2). Cell nuclei appear in red, while antibodies against BVDV are stained in green. When antibodies were present in the sample they bound to KOP-R cells infected with the CP7 isolate (**A**), while no green fluorescence could be seen when antibody-positive samples were tested against uninfected cells (**B**). Antibody-negative serum sample neither showed significant interaction with infected cells (**C**) nor with uninfected cells (**D**). Bar is 100 µm.

To ensure that no serum tested for *Pestivirus K* antibodies might be false positive due to interference with the *Pestivirus A* backbone, the sera were additionally tested against the BVDV cp7 virus isolate using KOP-R cells cultivated at the same settings as mentioned above for *Pestivirus F*, but with an incubation period of 72 h after cell infection. As a positive control sample, serum from an experimental BVDV-infected pig was used (Figure 4).

### 2.4. RNA Extraction and RT-PCR

All 1115 serum samples were additionally tested for viral RNA of BuPV and APPV by real-time RT-PCR. Therefore, 20 µL of five samples each were pooled and RNA was extracted using the King Fisher 96 Flex purification system (Thermo Scientific, Braunschweig, Germany) in combination with the MagAttract^®^ Virus Mini M48 Kit (Quiagen, Hilden, Germany) according to the manufacturers instructions. For APPV, a real-time RT-PCR was performed as described by Postel et al. [26], targeting the NS3 region, and for BuPV a real-time RT-PCR was performed as described by Dalmann et al. [27]. Sera of positive serum pools were then extracted as described above using 100 µL of each sample and again tested by real-time RT-PCR.

## 3. Results

A reliable differentiation between antibody-positive and -negative sera was enabled by both formats, the infection with culture grown virus (Figure 2) and the transfection of cells with the synthetic chimeric pestivirus replicon (Figure 3). The respective positive control serums showed stable positive results throughout all plates. All serum samples tested negative for antibodies against BVDV-1 strain CP7 (used *Pestivirus A* backbone virus) and the BuPV isolate (representing *Pestivirus F*). In addition, no viral RNA of BuPV was found in any serum sample.

In 182 (16.3%) out of the 1115 porcine serum samples, antibodies against APPV could be detected, and the positive sera originated from 51 of the 122 sampled farms (41.8%) (Figure 4, Table 1).

Overall, a seroprevalence of APPV-specific antibodies of 15.3% was determined in the 600 samples collected in 2009/10 and in 17.5% of the 515 pigs tested in 2018. In 46.7% of the 60 farms investigated in 2009/10 and in 37.1% of the 62 farms tested in 2018, anti-APPV antibodies were detected. In 2009/10, MV showed the highest seroprevalence in individual animals on state level with 24.2%, followed by ST (23.4%), BB (21.4%), BW (13.9%), and NI (4.5%), with no data available from BY and NW for these years. The interherd prevalence was highest in this period in ST (77.8%), followed by MV (70.0%), BW (42.9%), NI (30.8%), and BB (28.6%). In the year 2018, 27.1% of tested porcine sera collected in NI contained antibodies against APPV, and MV showed a seroprevalence of 26.7%, BW 23.9%, BY 18.7%, BB 8.6%, NW 8.3%, and ST 7.3%. Antibody-positive sera originated from 80.0% of tested farms in NI, 45.5% in BY, 44.4% in MV, 40.0% in NW, 28.6% in BW, 25.0% in ST, and 23.1% in BB.

On a farm level, there were farms where all tested animals had antibodies against APPV, as well as farms without a single positive animal. Two of the farms were included in the 2009/10 as well as in the 2018 sampling. Both farms tested positive in 2009/10. In 2018, antibodies could be again detected in one of the farms, while every sample collected at a second farm tested negative in this year (Table 2).

RT-PCR testing for the detection of viral APPV RNA revealed four positive serum pools. The following testing of single serum samples detected five positive samples overall—four in 2009/10 and one in 2018. Four farms contained at least one animal, in which viral RNA of APPV was detected (Table 2).

## 4. Discussion

Making use of the interchangeability of surface proteins within the quite diverse pestivirus group, it was possible to create chimeric viruses based on the well-studied *Pestivirus A* strain CP7 (backbone) and the virus in question, as it is shown here for *Pestivirus K* [25,28]. This concept could also assist early in epidemiological studies for other pestiviruses, which will be discovered or will emerge in the future and that challenge researchers continually with virus cultivation, as was the case for APPV [29]. By using chimeric pestivirus clones, seroprevalences may be investigated for new viruses as soon as the genetic information of the main immunogens is available. At this point in research, the detection of antibodies is the main goal. The presented method is able to detect antibodies without the use of a cell culture-adapted virus. Theoretically, even a single cell on the infected cell layer that emits a respective signal can prove the presence of binding antibodies.

E2 is the main surface protein of pestiviruses, normally inducing a profound serological response following infection, including neutralizing antibodies. Research on the humoral immune response against APPV shows that this is also the case for this newly discovered pestivirus [30]. Several E2 chimeric pestiviruses were shown to be able to replicate efficiently in susceptible cells [25,28]. If the pestiviruses are very distantly related, a combined E1 and E2 substitution could be necessary for the rescue of a recombinant chimeric virus [25]. In some cases, the chimeric construct is able to induce autonomous RNA replication, but these so-called replicons are not able to produce virus progeny. However, the important immunogens are still expressed by the replicons and the resulting antigens can be used for antibody detection. E1 itself is believed to be situated inside the virus envelope and, therefore, does not produce significant antibody response, but since it is partially included in the envelope in a heterodimeric complex with E2, a substitution of both E1 and E2 could be beneficial for the generation of an infectious chimeric virus. However, substitution of APPV E1 and E2 in the CP7 backbone was not sufficient for virus generation and, therefore, cells had to be transfected for the replicon-based expression of APPV E1 and E2. Transfection of cells with the replicons enabled mass production of test plates for the high number of serum samples that were tested and, therefore, ensured a consistent quality of all test plates. In addition, generation of replicons in a pestiviral background much better ensures the correct folding of the surface proteins, which is essential for a successful binding of antibodies. A transfection with plasmids for the E1 and E2 expression alone might lead to an incorrect three-dimensional structure that leads to an inactive/wrongly folded or presented protein and may result in false negative results.

Here, the focus was laid on ‘atypical’ porcine pestiviruses. An indirect immunofluorescence test was performed to detect anti-APPV antibodies by using a chimeric construct, while for the detection of anti-BuPV antibodies a classical cell culture isolate-based test principle was selected. So far, the search for virus of the species *Pestivirus F* remained negative outside of the initial endemic region in Australia [11]. Accordingly, all German samples analyzed in the present study reacted negative in the indirect immunofluorescence test as well as in the RT-PCR testing, further determining the lack of contact of the German pig population with this virus species. In contrast, antibodies against viruses of the species *Pestivirus K* seem to be widespread in Germany. This virus species is a supposedly important newly discovered pestivirus as it is linked to CT in newborn pigs and survey studies based on RT-PCR testing already showed the presence of viral genome in various countries around the globe. The first serological studies based on the testing of current samples by an in-house ELISA system demonstrated a higher seroprevalence in comparison to virus prevalences detected by RT-PCR in domestic pigs [17] or boar [31]. Even though the mentioned ELISA system is based on a different structural protein (E^rns^), both systems, i.e., the E^rns^-based ELISA and the E1/E2-based chimeric pestivirus, provided concordant results showing a high APPV-specific seroprevalence and only few samples that were positive for viral APPV RNA.

Moreover, APPV seems to have been circulating in Germany since 2009 and most likely even much longer as the overall seroprevalence in the years 2009 and 2010 was already at 15.3% and antibody-positive animals were present all over Germany. To determine the exact time point of *Pestivirus K* introduction into the pig population, much older samples need to be tested, presumably taken before the 1990s since the currently earliest detection of APPV in Europe dates back to 1997 in Spain [15] and even 1986 in Switzerland [32].

The high overall prevalence on farm level in both investigated time points additionally hints at the high pressure of infection in Germany, since roughly one out of three farms seems to have been in contact with the virus. However, in contrast to the generally comparable results of the two time periods, results on state level are quite diverse, which might be related to the described wave-like outbreaks of CT type II [20]. Since there is still only limited information available about the route of transmission, infection dynamics, spread, and pathogenesis of APPV [33] the reason for this certain outbreak pattern remains unknown.

In addition to a first estimation of the seroprevalence of APPV in Germany, 37 samples that tested positive for APPV-specific antibodies were examined more closely. The titration of these selected samples showed titers, ranging from 1:100 to 1:3200. A dilution of less than 1:100 is not common for indirect immunofluorescence testing, since it leads to an intense background signal. Serum samples with a low antibody concentration might, therefore, not be detected with this method. However, this is most likely not changing the classification of farms, since testing more animals would in most cases include clearly positive animals. Furthermore, the detected prevalences are high, which is also more indication that not too many samples are false negative. However, there are little data about the humoral response against APPV [30], therefore the evaluated titers cannot be put into perspective yet and an even higher seroprevalence of APPV in the German pig population might be possible.

## 5. Conclusions

In conclusion, a high overall seroprevalence of anti-APPV antibodies could be demonstrated in the German pig population, while BuPV seems to be still absent. Moreover, the presented test principle based on the generation of chimeric cDNA constructs might be applied for sero-epidemiological studies when further, hitherto unknown, pestiviruses emerge in the future.

## Figures and Tables

**Table 1:** Number of farms and individual animals tested per German federal state and percentage of farms and animals that tested positive for antibodies against APPV.

Federal State	Level	2009/10		2018				
		Number of Positive Samples/Total Number	% pos.	Number of Positive Samples/Total Number	% pos.		Number of Positive Samples/total Number	% pos.
Mecklenburg-Western Pomerania	farms	7/10	70.0%	4/9	44.4%	Σ	11/19	57.9%
	individual	24/99	24.2%	24/90	26.7%		48/189	25.4%
Lower Saxony	farms	4/13	30.8%	4/5	80.0%	Σ	8/18	44.4%
	individual	9/199	4.5%	16/59	27.1%		25/258	9.7%
Saxony-Anhalt	farms	7/9	77.8%	3/12	25.0%	Σ	10/21	47.6%
	individual	34/145	23.4%	4/55	7.3%		38/200	19.0%
Brandenburg	farms	4/14	28.6%	3/13	23.1%	Σ	7/27	25.9%
	individual	9/42	21.4%	9/105	8.6%		18/147	12.2%
Baden-Wuerttemberg	farms	6/14	42.9%	2/7	28.6%	Σ	8/21	38.1%
	individual	16/115	13.9%	16/67	23.9%		32/182	17.6%
Bavaria	farms			5/11	45.5%	Σ	5/11	45.5%
	individual			17/91	18.7%		17/91	18.7%
North Rhine-Westphalia	farms			2/5	40.0%	Σ	2/5	40.0%
	individual			4/48	8.3%		4/48	8.3%
Overall	farms	28/60	46.7%	23/62	37.1%	Σ	51/122	41.8%
	individual	92/600	15.3%	90/515	17.5%		182/1115	16.3%

**Table 2:** Number of animals that tested positive for antibodies against APPV in relation to overall tested animals of each farm sorted by year and German federal state. Both of the farms that were sampled at both time points are marked by color shading, one in red and the other one in blue. Farms, where viral RNA of APPV was detected by RT-PCR testing in at least one animal, are shaded in gray.

Federal State	Year	Number of Positive Samples/Total Number of Each Farm													
Mecklenburg-Western Pomerania	2009/10	9/10	5/10	3/10	3/10	1/10	1/10	0/10	0/10	0/10	2/9				
	2018	10/10	8/10	3/10	0/10	3/10	0/10	0/10	0/10	0/10					
Lower Saxony	2009/10	0/80	3/10	3/10	1/10	0/10	0/10	0/10	0/10	0/10	0/10	0/10	0/10	2/9	
	2018	0/25	3/15	7/10	3/5	3/4									
Saxony-Anhalt	2009/10	14/20	5/20	0/20	2/19	0/19	4/13	2/13	6/12	1/9					
	2018	2/5	1/5	0/5	0/5	0/5	0/5	0/5	0/5	0/5	0/5	1/3	0/2		
Brandenburg	2009/10	3/7	2/7	0/5	1/4	3/3	0/3	0/3	0/2	0/2	0/2	0/1	0/1	0/1	0/1
	2018	3/12	0/12	4/11	0/12	0/11	0/11	0/10	2/8	0/5	0/5	0/5	0/2	0/1	
Baden Württemberg	2009/10	3/10	1/10	0/10	6/9	2/9	1/9	0/9	3/8	0/8	0/8	0/7	0/7	0/6	0/5
	2018	2/25	14/15	0/19	0/2	0/2	0/2	0/2							
Bavaria	2018	1/9	0/9	0/9	0/9	0/9	1/8	6/8	0/8	4/8	5/7	0/7			
North Rhine-Westphalia	2018	3/14	0/14	1/10	0/9	0/1									

## Supplementary Materials

The following are available online at https://www.mdpi.com/2306-7381/6/4/86/s1, Table S1: Primer set used for plasmid construction.
