# Supplementary material for: Seroprevalences of Newly Discovered Porcine Pestiviruses in German Pig Farms
Source: Vet Sci. 2019 Oct 25;6(4):86. doi: 10.3390/vetsci6040086 (PMC6958323; doi:10.3390/vetsci6040086)
Supplement: Supplementary file 1 [file vetsci-06-00086-s001.pdf]

Appendix A

Table A1. Primer set used for plasmid construction.

Primer	Sequence (5′ to 3′)
**Ph_E1_APPV_F**	CATGGTTTGGGGCATATGCAATAGTACCATATTGCGAAACC
**Ph_E2_APPV_R**	TCACCTGCCCCATACTGGACACTAGCTTCCACCTGTAGAG
